# Novel and Alternative Therapeutic Strategies for Controlling Avian Viral Infectious Diseases: Focus on Infectious Bronchitis and Avian Influenza

**DOI:** 10.3389/fvets.2022.933274

**Published:** 2022-07-22

**Authors:** Ghulam Abbas, Jia Yu, Guangxing Li

**Affiliations:** Heilongjiang Key Laboratory for Animal and Comparative Medicine, College of Veterinary Medicine, Northeast Agricultural University, Harbin, China

**Keywords:** therapeutic agents, infectious bronchitis, avian influenza, herbal medicine, nanotechnology, infectious diseases control

## Abstract

The growth of poultry farming has enabled higher spread of infectious diseases and their pathogens among different kinds of birds, such as avian infectious bronchitis virus (IBV) and avian influenza virus (AIV). IBV and AIV are a potential source of poultry mortality and economic losses. Furthermore, some pathogens have the ability to cause zoonotic diseases and impart human health problems. Antiviral treatments that are used often lead to virus resistance along with the problems of side effects, recurrence, and latency of viruses. Though target hosts are being vaccinated, the constant emergence and re-emergence of strains of these viruses cause disease outbreaks. The pharmaceutical industry is gradually focusing on plant extracts to develop novel herbal drugs to have proper antiviral capabilities. Natural therapeutic agents developed from herbs, essential oils (EO), and distillation processes deliver a rich source of amalgams to discover and produce new antiviral drugs. The mechanisms involved have elaborated how these natural therapeutics agents play a major role during virus entry and replication in the host and cause inhibition of viral pathogenesis. Nanotechnology is one of the advanced techniques that can be very useful in diagnosing and controlling infectious diseases in poultry. In general, this review covers the issue of the poultry industry situation, current infectious diseases, mainly IB and AI control measures and, in addition, the setup of novel therapeutics using plant extracts and the use of nanotechnology information that may help to control these diseases.

## Introduction

Poultry production plays a vital role in food production and poverty alleviation in the absence of other nutrient-rich food items (1, 2). There are several important factors, such as poultry immunity, health, and production, which contest the future growth of the poultry industry (3). Globally, poultry diseases are continuously emerging to be the main subject in the poultry industry (4). Infectious bronchitis (IB), avian influenza (AI), Newcastle disease, and Gumboro disease are considered the common poultry diseases globally (5, 6). Although abundant consideration has been paid to limiting infectious diseases to avoid losses, these diseases continue to emerge and re-emerge (7). Avian infectious bronchitis virus (IBV), of the genus *Gammacoronavirus*, is one such kind of virus. In 1931, IBV was the first coronavirus identified in poultry and was considered a very vital pathogenic virus in livestock (8). At present, avian coronavirus IBV is a major economic pathogen of domestic poultry that causes mortality and significant losses in production regardless of vaccination (9). Many researchers have reported worldwide outbreaks caused by IBV in chickens and other birds that are characterized by high morbidity, mortality, and poor egg and meat production (10, 11). Currently, live attenuated vaccines are extensively used for the prevention and control of IB (12, 13). However, due to the higher diversity in viral genetic composition and the emergence of novel strains, the efficacy of vaccination is being greatly compromised (14).

Avian influenza virus (AIV) is another very important virus that belongs to the family *Orthomyxoviridae* and causes huge losses to the global poultry industry (15, 16). This virus has an eight-segment negative, single-stranded RNA and encodes about 11 proteins (17). In addition, AIV is divided into subtypes based on the hemagglutinin (HA) and neuraminidase (NA) proteins that are present on the surface and are responsible for virus attachment and release, respectively (18, 19). The reassortment of HA and NA with different subtypes might consequence in disastrous pandemics as H1N1, H2N2, and H3N2 in humans (20). Based on its pathogenicity, the virus is categorized into highly pathogenic AIV (HPAIV) and low pathogenic AIV (LPAIV) (21, 22). The co-circulation of many subtypes of AIV, such as H5, H7, and H9, makes vaccination unsuccessful for production (23). Thus, it is very important to develop a vaccine that can be useful for multiple serotypes. Considering these factors, the risk of emergence of novel serotypes and infectious diseases into the human population and livestock increases (24). For instance, the occurrence of the highly pathogenic H7N9 AIV caused huge losses due to the intensification of the poultry production system. The increased density of poultry stocks and upsurges in populations lead to higher transmission between birds and humans (25). Additionally, the increased rate of evolution and pressure on the immune system limit the effect of vaccination (26). Furthermore, the use of antibiotics in feed has raised the issue of drug residues and ultimately pathogen resistance, so that the subtherapeutic use of antibiotics has been totally banned in European countries since January 2006 (27).

Therefore, it is becoming very imperative to develop new strategies to control these types of diseases. For this purpose, consideration is being given to antiviral herbs that have no noticeable side effects on human and poultry health (28, 29). The influence of novel and alternative therapeutic agents on definite immune functions and a decrease in hazards might be very useful to counter viral infection (30). In the past, some derivatives and feed additives, i.e., plant extract, prebiotics, probiotics, enzymes, and yeast, have been reported to have immunomodulatory effects (31, 32). Their effects include improving metabolic status, decreasing physiological stress, inhibiting the expedition of cytokines by macrophages, and antimicrobial activity, thus enhancing immunity (33, 34). Antibodies (Abs) from mammals have been used for diagnostic and for therapeutic purposes against the invasion of pathogens (35), however, these Abs are obtained by invasive techniques. Thus, avian eggs have been considered for alternative production of Abs for therapeutic purposes against pathogen invasion (36, 37). Another important application, such as different nanomaterials that promote interactions between molecules and the virus, and enables researchers to construct a portable electroanalytical biosensing analyzer that effectively detects the virus (38, 39) along with the development of a nano-based viral vaccine. Generally, the development of first-generation vaccines has been produced by inactivation/killing or live attenuation of living organisms and second- and third-generation vaccines have been developed using RNA/DNA subunits (40, 41). Though subunit-based vaccines have many advantages, such as lower cost and the proficiency to produce an immune response against a specific pathogen as compared to conventionally developed vaccines (42), there are some disadvantages, such as poor immunogenicity, toxic effect, and are *in vivo* intrinsically instable along with multiple boosters. Hence, the development of a novel vaccine that can perform as an immunogen along with adjuvants to produce protection against the pathogen with enhanced immune response (43) and certainly nanotechnology is an advance technology that deals with the shortcomings of conventional vaccines.

Genomic assortment and outbreaks of such infectious diseases can turn into an epidemic and cause a widespread adverse effect on the global trade of poultry products along with human health (20, 44). The development of novel antiviral treatment strategies to control IBV and AIV is urgently required as existing inimitable tasks concerning its control in commercial poultry farming. Therefore, in the present review, we discuss possible new strategies and plant derivatives along with the use of nanotechnology that might be helpful to better control IB and AI.

## Medicinal Plant Derivatives Against IBV Infection

**Figure 1:**
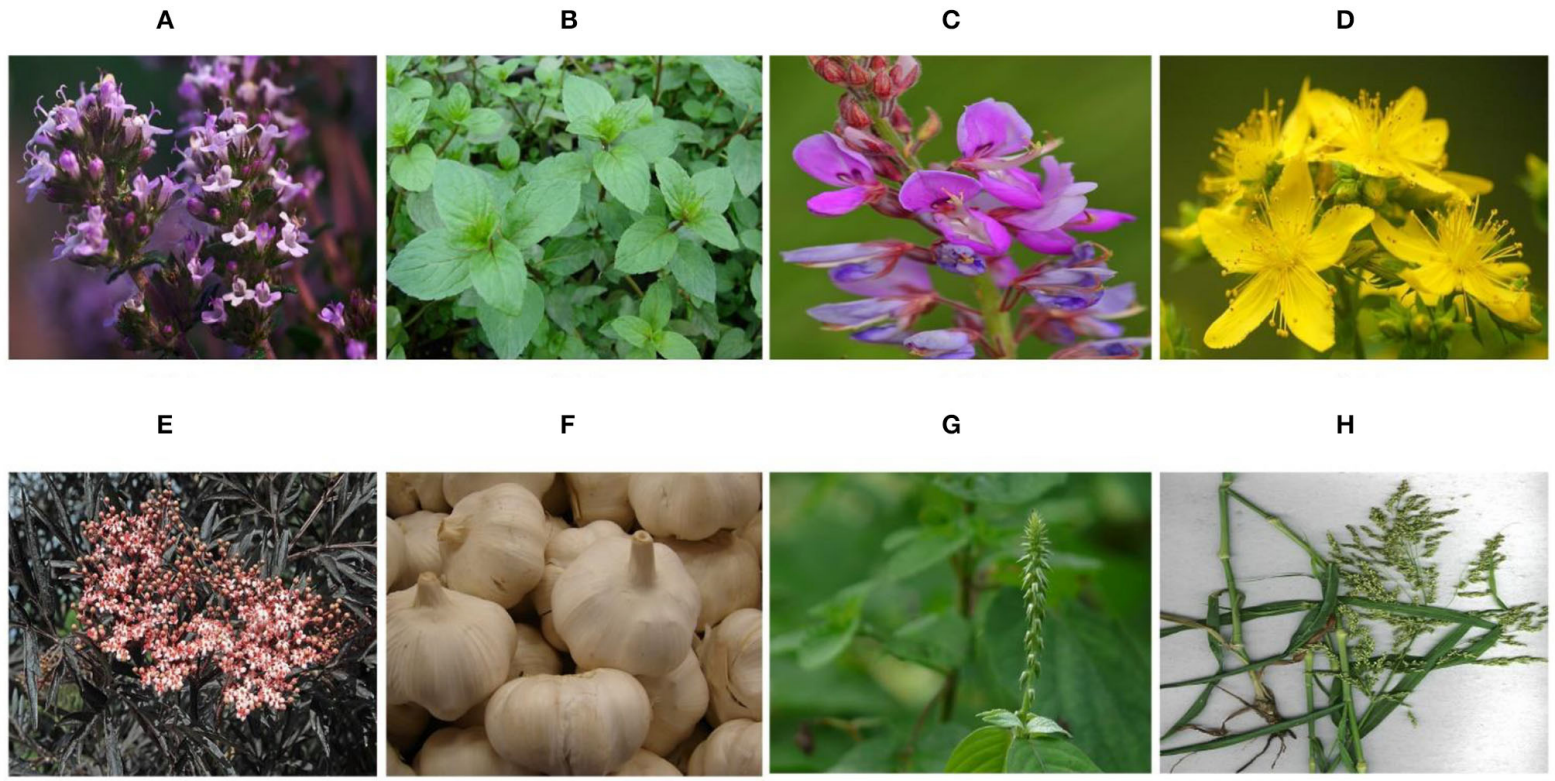
Plants in therapeutics to control infectious bronchitis (IB). **(A)** *Thymus vulgaris* (45). **(B)** *Mentha piperita* (46). **(C)** *Desmodium canadense* (47). **(D)** *Hypericum perforatum* (48). **(E)** *Sambucus nigra* (49). **(F)** *Alium sativum* (50). **(G)** *Achyranthes aspera* (51), and **(H)** *Panicum antidotale* (52).

Research on plant-derived antiviral substances is inadequate in comparison with the search for antimicrobial properties. However, a number of investigations have publicized the positive role of plant extracts in relation to antiviral effect (Figure 1). Assessment of different plant extracts was conducted, and *Thymus vulgaris, Mentha piperita*, and *Desmodium canadense* showed an antiviral effect against IBV preinfection and postinfection (53). *Hypericum perforatum L*, which is also recognized as Saint John's Wort, has been well studied for its biochemical composition and pharmacological activities (54). The extracts of *H. perforatum*, such as hypericin (HY), quercetin, pseudohypericin, and quercitrin, were assessed as antiviral against IBV. It was proved that a reduction in mRNA expression of pro-inflammatory cytokines [interleukin-6 (IL-6) and tumor necrosis factor-α (TNF-α)] *via* the nuclear factor kappa B (NF-κB) signaling pathway and upregulation of type I interferon *via* the MDA5 signaling pathway (55). Similarly, in another study, the natural compound of HY polycyclic quinone (56) has been proven to be antiviral against IBV infection in chicken embryonic kidney cells (CEK). IBV-infected CEK cells were treated with HY, and were found to upregulate anti-apoptosis genes such as Bcl-2 and to downregulate apoptosis-associated genes such as Caspase 3, Caspase 8, Bax and Fas, FasL, and JNK (57). The extract of *Sambucus nigra* was reported to have an inhibitory effect against IBV infection. When IB occurs at the early age of chicken,. *nigra* extract has shown inhibition of IBV infection in the early phase of disease infestation (58). Thus, it can be used for the inhibition of IBV and other CoVs. Garlic from the genus *Allium* of the family *Amaryllidaceae* was used as an antitherapeutic use in early times (59). The antiviral potential of garlic against IBV has been reported when exposed to various IBV strains and it showed an inhibitory effect (60). A*llium* (garlic) have been found to be a very effective therapeutic agent against corona viruses including COVID-19 as far as the immune system is concerned (61).

Similarly, the immune system of chicken against IBV has shown to be resistant during the supplementation of extract of sweet orange peel (62). Shahzad et al. (63) reported that various plants, such as *Achyranthes aspera, Neuroda procumbens, Panicum antidotale, Ochthochloa compressa, and Suaeda fruticose*, were very effective against all poultry viruses through their extracts, hence their use can be beneficial in future IBV infections. These plants extracts are useful in controlling the viral growth and have antiviral effect. The optimum antiviral activity and lowest viral growth were observed with the extracts of *S. icolados* and *O. compressa*, in terms of HA/HI as HA titer = 0 showed complete control over viral growth (64). The essential oils (EO) have been used for different viruses, such as dengue and herpesvirus, to assess virucidal activities (65, 66). Jackwood et al. (67) have carried out a research using two different hosts, i.e., Embryonated eggs (ECE) and Vero cells (E6). The EO designated as QR448a and botanical oleoresins in liquid emulsion was administered to the chicken infected with IBV. Following the aforementioned treatment, the clinical manifestation, pathological lesion, and RNA of IBV decreased, confirming the positive effect of EO and oleoresins.

## Use of Nanotechnology to Control IBV

**Figure 2:**
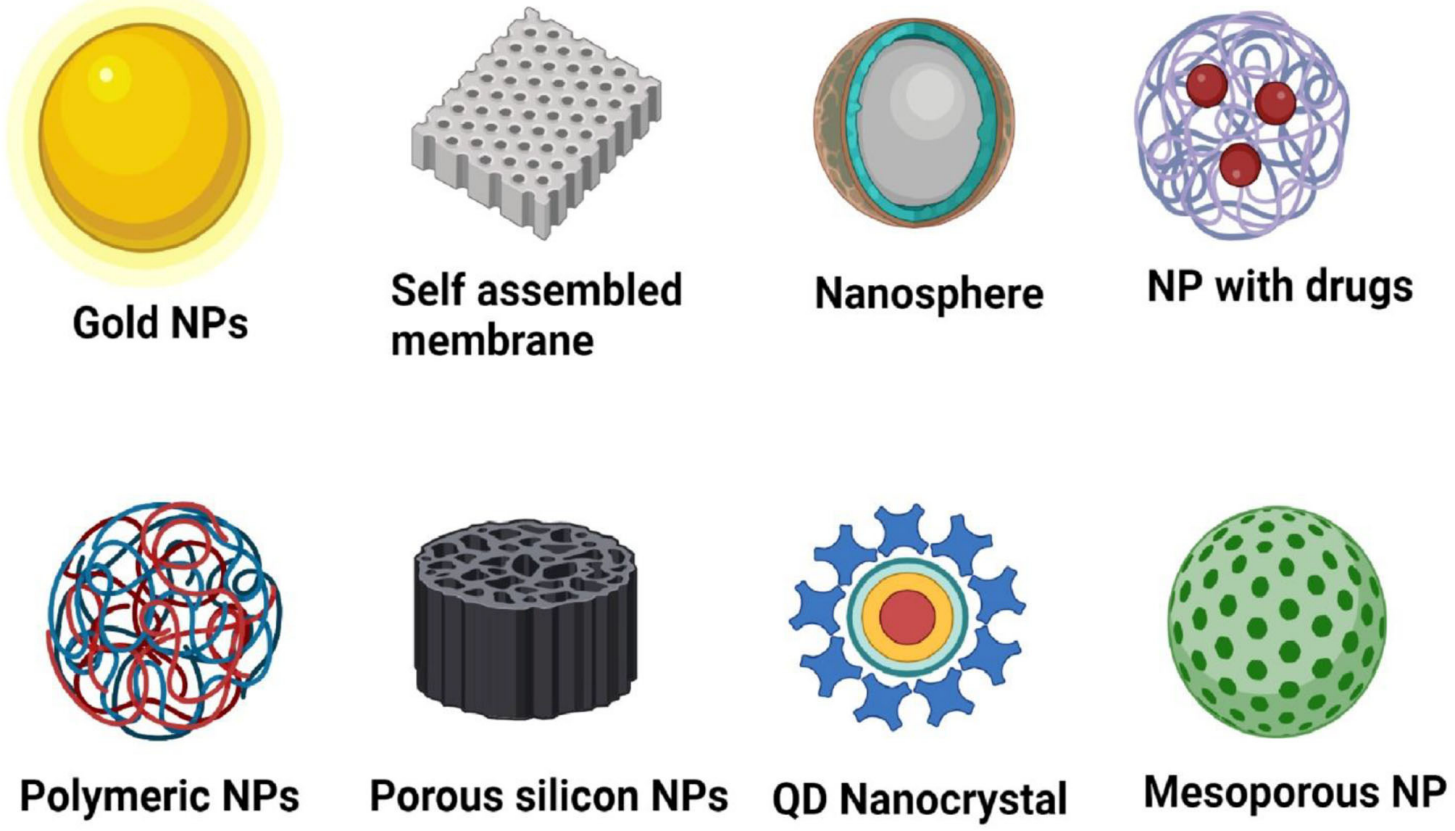
Different types of nanoparticles used in biomedicine for therapeutics of viral diseases. NP, Nanoparticles created with Biorender.com.

Nanotechnology is one of the inventive skills that have an inordinate possibility of uses along with a socio-economic prospective in the global poultry industry (68). The origin of nano is a word from the Latin “nanus” that mean a lesser, dwarf, or minute unit approximately 1 nm equals 10^−9^ m (69). Nanotechnology has developed the area of biomedical sciences, with a vast variety of NPs and capability in regard to diagnosis to therapeutics for viral infectious diseases (Figure 2) (70, 71).

The antiviral effects of G-Ag nanocomposites against IBV and Feline CoV (72) were investigated. Li et al. (73) have prepared a vaccine based on IBV-flagellin self-assembled protein nanoparticles (SAPNs) against IBV by incorporating spike protein as an adjuvant with the flagellin. IBV-challenged chicken with the aforementioned nano-vaccine showed a higher Ab response confirming its protective role. In another research, adjuvant-based nano-carriers of Quil-A and chitosan (QAC) having a size <100 nm were developed by Chandrasekar et al. (74). Furthermore, encapsulation with plasmid DNA (pQAC-N) vaccine and coding nucleocapsid (N) protein was done. When this vaccine was administered intranasally, improved immunogenicity and protection in terms of humoral and cellular immunity against IBV infection were observed. In addition, a decrease in viral load and reduced severity of clinical symptoms were observed. Polymeric carbonized nano-gels (CNGs) are also very effective in terms of IBV therapeutic agents as CNGs are very adsorbent on virus particles that may obstruct S1 and S2 glycoproteins to interact with host cells during infection. The development of CNGs is carried out at higher temperatures that possess a greater positive charge, which might cause the neutralization on the surface charge of IBV and weaken the pathogenicity of virus (75). Chou et al. (76) demonstrated the amalgamation of CNGs from lysine hydrochloride by a simple pyrolysis method, which resulted in the inhibition of virus against IBV. At a very lower concentration of 30 μg/ml, its efficiency was demonstrated by showing an inhibitory effect >98% in IBV-infected chicken embryos.

**Figure 3:**
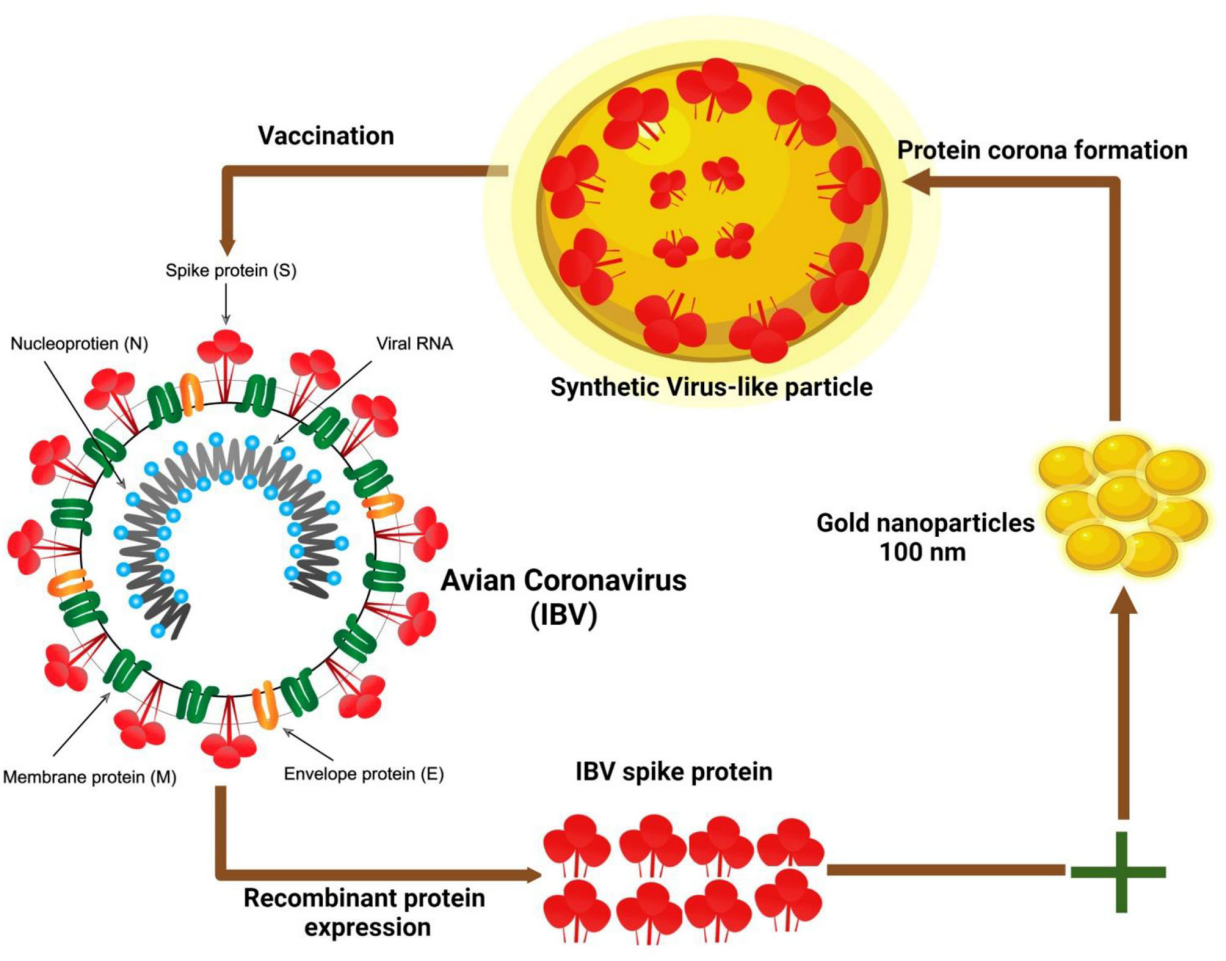
Schematic illustration of the preparation of synthetic virus-like particles (sVLPs) of avian corona virus. sVLPs were prepared from optimized mix enclosing spike (S) protein of infectious bronchitis virus (IBV) and 100 nm gold nanoparticles through impulsive development of corona protein (72).

Several uses of full NP based on magnetic and gold quantum dots (QDs) for virus detection and tracing have been described (77). Ahmed et al. (78) reported a novel method by linking anti-IBV Abs with QDs for the production of an immune-link of chiral-QDs as a chiro-immuno-sensor for IBV from blood samples of chicken. Furthermore, a self-assembled nanostructure was established for the limit need to be used for detection and 47.91 egg infection dose (EID)/50 ml, was quite efficient in examination of the target virus (79). Virus-like particles (VLPs) have been extensively studied and developed to transport a range of compounds, including medicines, peptides/proteins, RNA/DNA, Abs, and vaccines, for use as antigen nanocarriers and adjuvants to immune cells in an attempt to elicit a protective humoral immune response (80). Cell surface protein S that binds to the receptor can induce the body to produce an immune response (81). Chen et al. (82) have described a classic approach to the use of avian CoVs VLPs-based S protein using 100-nm gold np incubation with an optimized concentration of viral proteins (Figure 3). The aforementioned study resulted in the impulsive development of proteins with the induction of the assembly of virus-like nanostructures with viral antigens coating the fundamental particulate. Furthermore, it was concluded that the results of VLPs from the present study validate the successful preparation of synthetic VLPs (sVLPs) through NP, innate inclination to persuade protein coating (83, 84).

## Use of Virucidal Drugs Against IBV Infection

Advanced studies in the molecular biology of viruses have highlighted many potential targets for antiviral drugs (85). The efficacy of virus activity limiting drugs, such as Argovit, Triviron, Ecocid, and lauric acid monoglycerides, has been tested in chickens by inoculation of the vaccine strain of IBV H120 against IB of chickens. These virucidal drugs were shown to possess potential virucidal activity in the small intestine against IBV (86). Traditional medicines, mainly from China, have been very effective against respiratory viruses (87). A combination effect of traditional Chinese medicines (TCMs), such as Shegandilong (SGDL) and doxycycline, was examined by Feng et al. (88) to prevent viral infection, and the outcomes indicated an increased level of tracheal immunoglobulin A (IgA). Additionally, SGDL granule and doxycycline efficiently subdued the replication of IBV and inhibited the propagation of IBV tropism between the trachea and lung. Furthermore, they controlled the expression of mRNA of IL-6, IL-1β, IFN-γ, and TNF-α, and reduced histopathological lesions of respiratory organs such as trachea and lung. In other similar studies, based on the combination of *glycyrrhizin diammonium* (GD) and lithium chloride (LiCl) conducted by Li et al. (89), IBV was used to infect cells. The effect of the drugs to inhibit the virus was established by using CEK. Additionally, the apoptotic effect was positively associated with the cytopathic effect and might be repressed in effect of the drug treatment. The replication of IBV and the effect of LiCl were examined in two different cells types, i.e., Vero cells derived from African Green monkey kidney-epithelial cells and DF-1 cells derived from chicken embryo fibroblast cells. The concentration of viral RNA and proteins was reduced after the aforementioned cells lined were treated with LiCl (90).

Amino acid derivatives are known for their antiviral ability against different poultry, animal, plant, and human viruses (91). The modification of amino acids with respect to antimicrobial peptides activities plays an important role in antiviral activities (92). The evaluation of antimicrobial peptides was carried out in terms of swine intestinal antimicrobial peptides (SIAMP) against IBV in chicken embryos. Pre-treatment of embryos with SIAMP and infected with IBV showed a remarkable reduction in mortality. Though the authors of this study did not present the characterization of the antiviral mechanism, they suggested that SIAMP might play a role during virus attachment to the cell surface, thus limiting IBV infection (93). Mannose binding lectin (MBL) is well known as an antiviral agent (94). In terms of IBV, recombinant chicken MBL showed antiviral activity during the direct interaction with virus particles that subsequently inhibited IBV infection (95). Thiazolidines are reported as antiviral inhibitors (96). The substitution of 2-aryl thiazolidine-4-carboxylic acids 1a-h was better antiviral than their N-acylated derivatives 2a and 3a, which would be promising antiviral agents against AIV H9N2 and IBV infections in the near future (97). Avian eggs can produce Abs that protect against pathogen entry by non-invasive techniques. Similarly, when Tsukamoto et al. (98) produced IgY (Ab) from ostrich eggs and used against IBV-infected chickens, infection was remarkably inhibited.

## Medicinal Plant Derivatives Against AIV Infection

**Figure 4:**
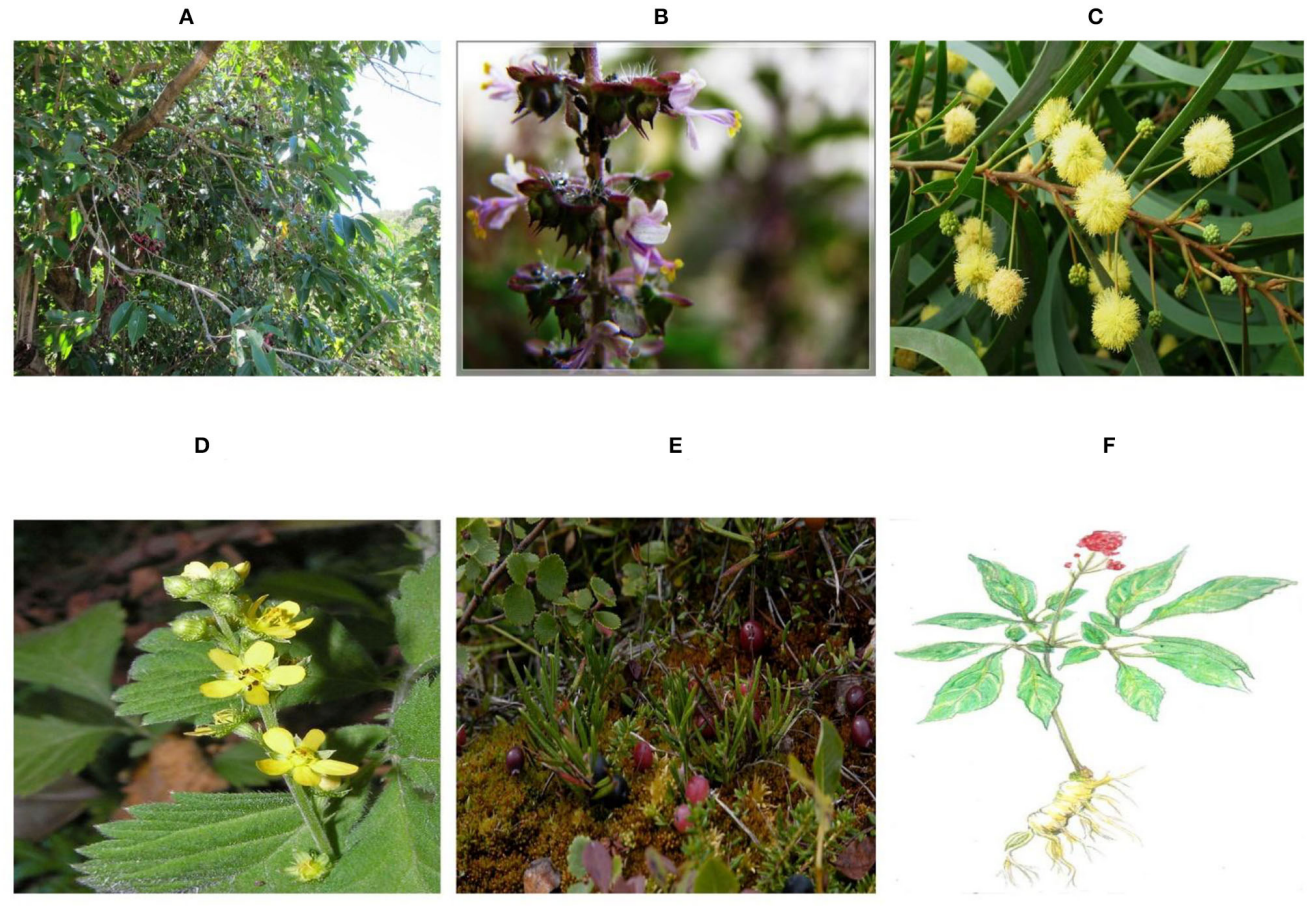
Plants in therapeutics to control avian infectious influenza. **(A)** *Eugenia jambolana* (99). **(B)** *Ocimum sanctum* (100). **(C)** *Acacia Arabica* (101). **(D)** *Agrimonia pilosa* (102). **(E)** *cranberry* (103), and **(F)** *Ginseng* (104).

Novel anti-influenza therapeutic techniques are a prerequisite for its control in the present era, and these developments can be achieved through the search for new ways to modulate the viral mechanism and immune system (Figure 4) (105–107). Numerous herbal species with potential inhibitory effects on the replication of influenza viruses were frequently described using *in vitro* cell culture methods and embryonated eggs or *in vivo* mouse models (108, 109). Many phytochemicals of relatively low molecular weight, such as polyphenols, flavonoids, terpenes, glucosides, and alkaloids extracted from different plants have been shown to be endowed with an antiviral activity against AIV (110, 111). In this regard, polyphenol-enriched extract of *Rumex acetosa* employs an inhibitory effect against AIV replication by weakening the attachment of viral particles to target cells (112).

The antiviral effect of the water extract of *Psoraleae semen* (WPS) has an auspicious role of novel anti-influenza. Choi et al. (113) have conducted a comprehensive study using RAW 264.7 and MDCK cells to assess the inhibitory effect of virus using 100 μg/ml in WPS. It was proven that WPS served as an immunomodulator and inhibitor of influenza HA and NA. Furthermore, they suggested that WPS can be a substantial alternativee as an antiviral therapeutic agent due to the disruption of infection *via* type I IFN-mediated signaling pathway involving RAW 264.7 cells. Similar findings were reported in which the anti-influenza effects of WPS produce direct inhibition of HA and NA mediation. The predominant role of Psoralen and bakuchiol in influenza and herpes simplex viruses was reported in WPS antiviral activity against many (114, 115). Recently, bakuchiol was found to exert anti-influenza viral activity *via* the activation of erythroid 2-related factor 2 (Nrf2) (116).

Oxidative stress tempted by infections of RNA virus may contribute to numerous features of viral disease pathogenicity including apoptosis, impairment of immune system, inflammation response, and body weight loss (117). One of these compounds, Yi-Zhi-Hao pellet (CYZH), is a known TCM that is used as an antiviral. Further, it induced the activation of Nrf2 and NF-κB, which subsequently upregulated heme oxygenase-1 (HO-1) expression. Also, CYZH protected cells from oxidative damage induced by reactive oxygen series. In conclusion, CYZH inhibits IAV replication *in vitro*, at least partly by activating the expression of the Nrf2/HO-1 pathway (118). Considering multiple components in CYZH, the other relevant mechanisms of anti-IAV are still needed to be considered in future study. Activation of the Nrf2/ARE pathway induces the expression of anti-inflammatory and anti-oxidative genes, such as HO-1, which is known to play a role in alleviating oxidative stress and tissue protection (119, 120). For example, previous studies have indicated that Nrf2 protect cells from the cytopathic effects of AIV, most likely by increasing the expression of antioxidant genes in human alveolar epithelial cells and modifies AIV entry and replication in nasal epithelial cells (121, 122). This mechanism further need to be studied in avian species in the future.

Sood et al. (123) found that extracts of *Eugenia jambolana* had a 100% virucidal effect against HPAIV H5N1 in tissue culture and ECE in ovo inoculation. In another study (124), *Ocimum sanctum* and *Acacia Arabica* crude extract and terpenoid isolated from the leaves of *O. sanctum* and polyphenol from *A. Arabica* have shown promising antiviral properties against H9N2 virus, showing significant virucidal activity. Future investigations are necessary to formulate combinations of these compounds for a broader antiviral activity against H9N2 viruses and evaluate them in chickens. The extracts of these plants were used on tissues in ovo, however; further study is needed in avian species. The extract of *Agrimonia pilosa* also exhibited a virucidal effect at a concentration of 160–570 ng/ml against influenza A and B viruses when the viruses were treated with the extract prior to plaque assay (125).

Luganini et al. (126) conducted research considering the direct action of **c**ranberry extract against influenza virus and showed that the novel Oximacro of cranberry extract impedes two subtypes (A and B) of influenza viruses. During *in vitro* studies, they elaborated on the mechanism of Oximacro that prevented virus entry and attachment, leading to virucidal activities. In the past, the immune response, mainly humoral, in chickens during the inactivation of the vaccine for AIV has been induced by the addition of immunoadjuvants and extracts of plants in feed. Similarly, when the stems and leaves of *Ginseng saponins* were added to the drinking water, the humoral immune response increased to a significant level. In another such instance, serum Abs level enhancement was observed significantly when *H. perforatum L*. was administered orally and in the drinking water (127, 128). When the extract of ginseng after fermentation was administered to mice, the protection level against different subtypes (H1N1, H3N2, H5N1, and H7N9) of the influenza virus. Additionally, components of the adoptive immune system such as B cell, CD4, CD8, and major histocompatibility complex II were observed. However, detailed studies are needed in the future to explore the anti-influenza mechanism of ginseng in fermented form (129).

The efficacy of *Sargassum pallidum* polysaccharides (SPP) as adjuvant in inactivated vaccines of NDV, AIV, and IBV in chickens was tested by Li et al. (130). In that study, the vaccines containing 10, 30, and 50 mg SPP/ml were compared with the traditional oil adjuvant vaccines. Serum Ab titers against the three viruses significantly increased at the dose 30 mg/ml. Moreover, the CD4 content and T lymphocyte multiplication were enhanced in all treated groups.

## Use of Nanotechnology to Control AIV

**Figure 5:**
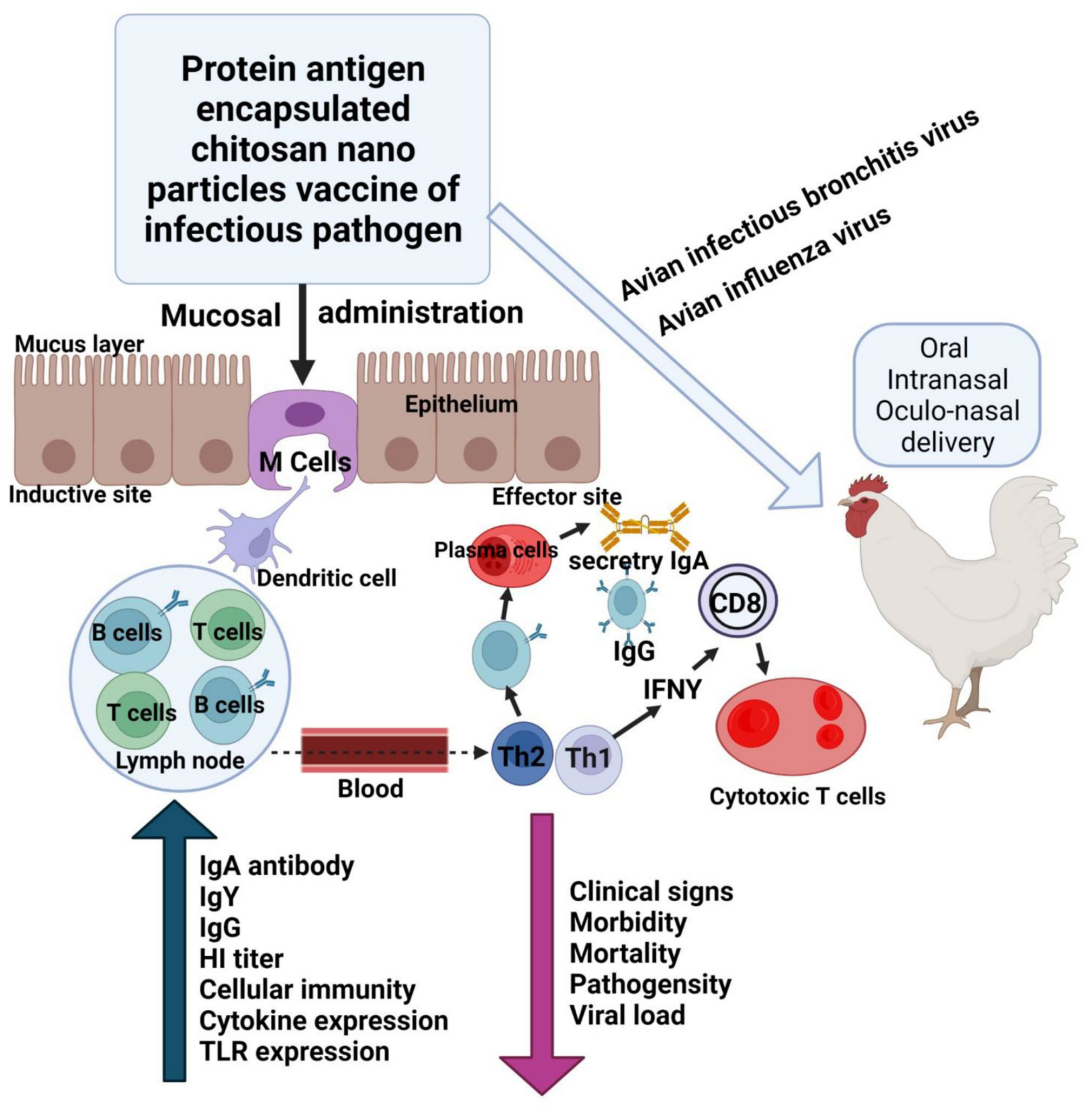
Schematic illustration of the use of nanoparticles (chitosan) to deliver vaccine during IB and avian influenza to mucosal sites to induce immune response. The modified figure was redrawn with permission from Renu and Renukaradhya (133). Image created with Biorender.com.

The application of various nanomaterials endorses the interactions between the materials and the virus, enabling other researchers to build a biosensing analyzer that should work effectively based on the portable electroanalysis, and perform exact detection of influenza virus (131). The use of nano-based vaccines has many advantages that include higher storage time, and the encapsulation of vaccines in NP that are polymers in solid form might assist to stabilize at room temperature, consideration of an alternate route of administration, and enabling precise discharge. Nano-vaccines might release soluble antigens that induce both types of immunity, i.e., humoral and cellular (132). In a similar way, NPs based on a chitosan derivative are used to deliver an immune response when administered into mucosal sites of poultry (Figure 5). Furthermore, a decrease in morbidity, mortality, and viral load is observed in chickens infected with IBV and AIV (133). Magnetic beads are also considered as nano beads and are used to identify signals at amplifion along with quartz crystal microbalance (QCM) apta sensors, the magnetic nano bead-amplified QCM immune-sensors have been used for the detection of the H5N1 protein (134). Silver nanoparticles (AgNPs) magnetic particles, and carbon-based materials are commonly used to analyze and identify the different subtypes of influenza viruses. The preparation of these particles is carried out based on the methodology already available in the literature (135, 136). Additionally, there are well-known techniques such as electrode-based well array, electrochemical quantitative systems, and on-chip nanomembrane tubular sensors of based on full integration (137, 138). Furthermore, mesoporous silica NPs performing functions with the amino group and naturally loaded with prodrugs of quercetin and shikimic acid discovered a novel antiviral nanoformulation that targets the detection of highly pathogenic avian influenza H5N1 virus. They induced also strong immunomodulatory effects: they limited the production of cytokines (IL-1β and TNF-α) and nitric oxide (NO) by 50%. Furthermore, it played an extraordinary role in the critical carrageenan-induced rat model to induce the antiinflammatory influence carried *in vivo* (38, 139). Thus, nanotechnology through the use of a variety of NPs and in the form of nano-vaccines, nanobodies and nanomedicine, along with the use of adjuvants, has a remarkable future perspective in the field of biomedicine to control avian infectious diseases.

The control of IBV and AIV has faced many troubles due to several factors such as drug resistance, emergence of novel viral strains, and cross-species infections. Plant extracts can be a potential source to develop novel therapeutic medicine. The use of nanotechnology in terms of nano-vaccines, nanobodies, and nanomedicine might be very helpful to diagnose and control these poultry infectious diseases. In the future, the exact mechanism of action to counteract poultry infectious diseases caused by novel viral strains needs to be studied to develop control strategies.

## Author Contributions

Conceptualization and writing—original draft: GA. Writing—review and editing: GA, GL, and JY. Supervision, project administration, and funding acquisition: GL. Validation: GL and JY. All authors agreed to the final version.

## Funding

The National Natural Science Foundation of China under Grant (31172295 and 31272569) supported this research.

## Conflict of Interest

The authors declare that the research was conducted in the absence of any commercial or financial relationships that could be construed as a potential conflict of interest.

## Publisher's Note

All claims expressed in this article are solely those of the authors and do not necessarily represent those of their affiliated organizations, or those of the publisher, the editors and the reviewers. Any product that may be evaluated in this article, or claim that may be made by its manufacturer, is not guaranteed or endorsed by the publisher.
